# Frequency selectivity of echo responses in the mouse primary auditory cortex

**DOI:** 10.1038/s41598-017-18465-w

**Published:** 2018-01-08

**Authors:** Meng Wang, Ruijie Li, Jingcheng Li, Jianxiong Zhang, Xiaowei Chen, Shaoqun Zeng, Xiang Liao

**Affiliations:** 1 Britton Chance Center for Biomedical Photonics, Wuhan National Laboratory for Optoelectronics, Huazhong University of Science and Technology, Wuhan, 430074 China; 2 Brain Research Center and State Key Laboratory of Trauma, Burns, and Combined Injury, Third Military Medical University, Chongqing, 400038 China; 3 CAS Center for Excellence in Brain Science and Intelligence Technology, Shanghai Institutes for Biological Sciences, Chinese Academy of Sciences, Shanghai, 200031 China

## Abstract

In the primary auditory cortex (A1), neuronal ensembles are activated relative to anticipated sound events following rhythmic stimulation, but whether the echo responses of the neurons are related to their frequency selectivity remains unknown. Therefore, we used *in vivo* two-photon Ca^2+^ imaging to record the neuronal activities in the mouse A1 to elucidate the relationship between their echo responses and frequency selectivity. We confirmed the presence of echo responses in a subgroup of mouse Layer 2/3 A1 neurons following a train of rhythmic pure tone stimulation. After testing with a range of frequencies, we found that these echo responses occurred preferentially close to the best frequencies of the neurons. The local organization of the echo responses of the neurons was heterogeneous in the A1. Therefore, these results indicate that the observed echo responses of neurons within A1 are highly related to their frequency selectivity.

## Introduction

When humans and other animals interact with the natural environment, the use of sensory cues and previous experience are critical to form predictions that guide behavior and ultimately survival in the world^1,2^. Recent studies have shown that anticipatory events are found at the levels of both brain activity and behavior. Sumbre *et al*. found that a zebrafish larva was capable of estimating the timing of a specific impending stimulus through repetitive visual stimulation^3^. Located in the caudal part of the auditory cortex, the primary auditory field (A1) displays a regularly tonotopic layout with low best frequencies positioned caudally and high best frequencies dorsoventrally^4^. The neuronal activities in the mouse A1 have also been found to contribute coding sound information and driving motor responses to expected events^5^. These studies have shown that repetitive stimulation at a regular inter-stimulus interval (ISI) can result in neuronal activity at the anticipated time interval after stimulation, a phenomenon known as “omitted stimulus-evoked responses” or “echo responses”^5–11^. Although the prediction correlated neural signals have already been observed in multiple sensory regions in different mammals, the stimulus type for the preferentially evoked echo responses is unclear. Specifically, whether the echo responses of the neurons in the auditory cortex are related to their frequency selectivity remains unknown.

The processing and representation of behaviorally relevant information have been suggested to be selectively enhanced by predictive sensing in an efficient manner. For example, a recent nonhuman primate study demonstrated that high excitability was confined both in time and across neuronal ensembles in the auditory cortex when auditory stimuli were predictable in both timing and pitch^12^. As spectro-temporal filters, the features of the responses in the brain were restricted in both time and space, which is an efficient strategy to minimize the energy expenditure required to maintain a specific cortical state^13,14^.

In the auditory cortex, the response profiles of neurons have often been shown to characterize sound features such as sound intensity and frequency^15–17^. Electrophysiological recordings and large-scale imaging techniques have revealed the global tonotopic map in auditory cortex and the coarse division of the separate fields in the cortex^18,19^. Although large-scale recording techniques normally demonstrate smooth tonotopic organization of the neuronal response patterns, the response properties of neurons may vary remarkably, even in neighboring neurons^17,20^. By elucidating the response properties of neurons to sound frequencies in a small brain region, the local populations in the A1 are revealed to be highly heterogeneous, which means that neighboring neurons may present similar or extraordinarily different frequency tunings properties^21,22^. Therefore, these studies demonstrate that a rough tonotopic organization emerges at large scales, but local heterogeneity emerges at fine scales (<250 μm) and is embedded in the global tonotopic order^22^.

The two-photon microscopy combined with Ca^2+^ imaging has allowed simultaneously sampling the activity of hundreds of neurons in the intact brain at cellular resolution^23,24^. Compared to electrophysiological recordings, two-photon Ca^2+^ imaging has less tissue damage and is able to detect subthreshold neuronal activity^23,25^. Furthermore, two-photon Ca^2+^ imaging can readily monitor activity of specific types of cells and precisely locate their positions within neural circuits^24^. For these reasons, *in vivo* two-photon Ca^2+^ imaging provided us with a powerful tool for searching for the population of neurons in A1 participating in the processing of expected sound events. Issa *et al*. previously used a genetically encoded Ca^2+^ indicator, GCaMP3, to address the issue of topographic organization at multiple scales in the A1^26^. Bandyopadhyay *et al*. used the synthetic indicator OGB-1, the most commonly-used Ca^2+^ indicator dye, to study the activity of individual neurons in the A1 during sound stimulation^25^. However, both of these indicators fail to detect Ca^2+^ transients caused by single spikes in living animals because of insufficient sensitivity^27–30^. The sensitivity plays the most important role in studying the organization of the A1, because most of cortical neurons respond with only one or two spikes to sound stimulation^20^. In contrast, recent studies showed that Cal-520^31^ with a high sensitivity was a more suitable Ca^2+^ indicator for functional mapping experiments in the A1^32^ of living brains.

Since *in vivo* two-photon Ca^2+^ imaging has become widely used for the study of the functional organization of neuronal populations in the cortex^22,25,33^, we applied this technique to investigate the relationship between the echo responses of the neurons and their frequency tuning in the A1. As stable two-photon imaging can be easily achieved under anesthesia, we initiated our experiments using anesthetized preparations. However, it has been suggested that general anesthesia can affect many aspects of spontaneous activity and sensory processing in the cortical circuits^34,35^. To confirm whether anesthesia could also affect predictive information processing in our conditions, we repeated these experiments in awake mice. Similar to broadband noise stimulation, as reported previously^5^, following a train of rhythmic pure tone stimulation, we found that a subgroup of mouse Layer 2/3 (L2/3) auditory cortex neurons showed one or multiple times of the echo responses at the anticipated time interval in the absence of sound. Testing with a series of pure tone stimuli, we revealed that these echo responses were typically elicited following stimulation at the neurons’ best frequencies. The local organization of the echo responses of the neurons was heterogeneous.

## Materials and Methods

### Animals

C57BL/6J mice (male, 2–3 months old) were supplied by the Laboratory Animal Center of the Third Military Medical University. These mice are a suitable and common animal model for auditory experiments^25,36^. All experimental protocols were carried out based on institutional animal welfare guidelines with approval of the Third Military Medical University Animal Care and Use Committee. The mice were housed in a cycle of 12 h light/dark (lights off at 19:00), free of accessing food and drinking water. In our experiments, 8 mice (777 neurons) and 4 mice (194 neurons) were used for Ca^2+^ imaging in anesthetized and awake conditions, respectively.

### *In vivo* two-photon Ca^2+^ imaging and electrophysiology

The right A1 was exposed to two-photon imaging in anesthetized mice as elsewhere^21,37–39^. In brief, we applied isoflurane at the level of 1–2% in pure oxygen for anesthesia and placed the animals onto a heating plate at a temperate of 37.5–38 °C. After local anesthesia with lidocaine, we removed the skin and muscle over the A1. Then the skull was glued with a customized plastic chamber using cyanoacrylic glue (UHU) and a ~2 × 2 mm small craniotomy was made centered at 2.5 mm posterior to bregma and 4.5 mm lateral to midline. After that, we reduced the isoflurane level to 0.4–0.8% and transferred the animal to the imaging system. The multi-cell bolus loading procedure with Cal-520 AM was similar to the previous studies^23,31^. The head fixation and training procedures were similar to our recent studies^5,32^. When performing two-photon imaging in awake mouse, an infrared camera (frame rate: 30 Hz) was used to collect the videos of the mouse behavior. Two-photon imaging was carried out with a resonant scanner-based “LotosScan 1.0” system (Suzhou Institute of Biomedical Engineering and Technology), as described in details previously^40,41^. To conduct targeted *in vivo* whole-cell recordings in neurons of A1, we used the previously described “shadow-patching” procedure^37–39,42,43^.

### Retrograde tracing

To verify that the imaged cortical regions were located in the A1, we used the criterion^44,45^ that the ventral part of the lateral medial geniculate body (MGBv) is connecting with A1. Hence, we first determined the center of the craniotomy window in a stereotactic way (70% of bregma-lambda and ventral: ~2 mm, or lateral: ~4.4 mm)^21,25^, and then performed *post hoc* histology with all mice after imaging experiments. For labeling cortico-thalamic projections, we used a glass electrode, which has a tip diameter of 20–30 μm, and filled it with neural tracer solution. In the experiment, we inserted the electrode into the cortical region at a depth of ~500 μm below the surface. We used Alexa Fluor 488-conjugated cholera toxin subunit B (CTB) as the neural tracer, and injected the fluorescent CTB solution with 0.5% in phosphate buffer by pressure (700 mbar) for 15 min. Seven days after the fluorescent CTB injection, the mice were anesthetized with pentobarbital (1.0 g/kg ip). The brain was first dissected out and then it was immersed with 4% paraformaldehyde overnight. To visualize fluorescent tracers, a consecutive series of coronal or horizontal sections (40 μm thick) were prepared using a sliding cryotome, and then all sections were mounted onto glass slides and imaged with a stereoscope (Olympus).

### Auditory stimulation

As shown previously^37^, the sounds were presented through a free-field ES1 electrostatic speaker and an ED1 speaker driver (Tucker Davis Technologies). The distance was 6 cm between the speaker and the contralateral ear of the mouse. The tone duration we applied in this experiment was 100 ms, containing 10 ms on and off linear ramps. To test the frequency tuning properties of single neurons, 11 frequencies logarithmically spaced in the range of 2–40 kHz were used in our experiment. Every frequency of stimulation was repeated 20 times, with the inter-stimulus interval (ISI) of 2 s.

All sound levels were measured with a microphone placed ~6 cm away from the speaker. The sound levels were calibrated by a pre-polarized condenser microphone (377A01 microphone, PCB Piezotronics Inc) as published previously^46^. The data were recorded at 1 MHz by using a data acquisition device (USB-6361, National Instruments) and then analyzed by our customized LabVIEW program. During pure tone stimulation experiments, the use of the sound level was equal to the levels of ~79 dB SPL (1 kHz to 10 kHz) and ~81 dB SPL (10 kHz to 40 kHz). The major components of the background noise (~60 dB SPL) in the experiments were low-frequencies, similar to the equipment used previously^37,38^. The peak of background noise, with a spectral density about 33 dB/sqrt(Hz), is located below 1 kHz. This component was out of the hearing range of mice (from 1 kHz to about 100 kHz)^47,48^ and therefore should not be concerned, as also we described in our previous studies^5,32^.

### Data analysis

We analyzed our data with software including MATLAB 2014a (MathWorks), Igor Pro 5.0 (Wavemetrics) and LabVIEW 2014 (National Instruments). We identified individual neurons in two-photon imaging data and performed the regions of interest (ROIs) drawing for each of them. In each image frame, fluorescence changes (f) were calculated by averaging the image intensities within each ROI. The 25th percentile of the entire fluorescence changes in single-trial was calculated as the baseline f0, and then neuronal Ca^2+^ signals were expressed as the relative changes of fluorescence Δf/f = (f − f0)/f0.

As described in our previous studies^5,32^, Ca^2+^ transient detection was performed based on thresholding criteria about peak amplitude and rising rate, which is similar to previously described peeling algorithm^49^. For the analysis of frequency tuning, we constructed the frequency tuning curves of individual neurons, and performed curve normalization and averaging as previously described^37^. To improve visibility here, the frequency tuning curves were fitted by Gaussian function.

Summarized data are presented as mean ± standard error of the mean (SEM) in figures. We used Wilcoxon signed rank test and Wilcoxon rank sum test to determine statistical significances for paired and unpaired cases, respectively. P < 0.05 was considered statistically significant.

### Data availability

The data that support the findings of this study are available from the corresponding author upon reasonable request.

## Results

### Spontaneous and pure tone-evoked responses in A1 L2/3 neurons

**Figure 1:**
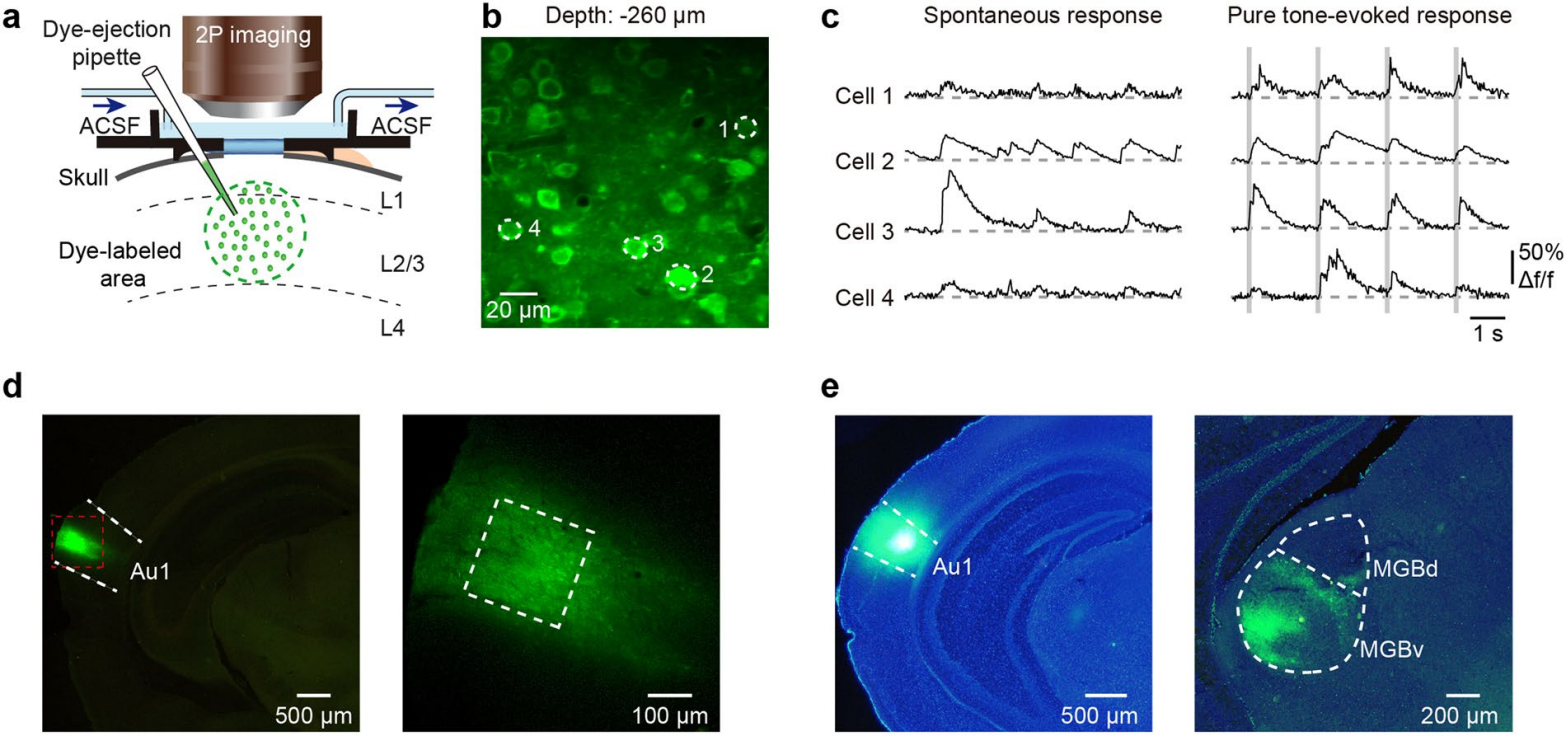
Spontaneous and sound-evoked activities of A1 neurons in anesthetized mice. (**a**) Side view of the experimental arrangement. (**b**) Two-photon image of Cal-520 AM-labeled neurons at 260 μm depth below the pial surface in an anesthetized mouse. Four exemplary neurons are labeled 1 through 4. (**c**) Four representative neurons marked in panel b show spontaneous (left) and sound-evoked activities (right). The vertical gray bars indicate tone stimuli. The tone frequency we used in the example was 4913 Hz. (**d**) Coronal slices show the injection site of Cal-520 in mouse A1 (left) and an enlarged image (right) of the region marked with a red box (left). The regions marked with a white box were imaging regions (right). (**e**) Coronal slices show the injection site of CTB-green, i.e., the retrograde neuronal tracer, in the A1 (left) and the retrograde-labeled cortical projecting neurons mainly in the lateral MGBv (right).

We used two-photon Ca^2+^ imaging^23^ in combination with the fluorescent Ca^2+^ indicator Cal-520 AM^31^ to measure the activity of L2/3 neurons in the A1 in isoflurane-anesthetized mice (Fig. 1a). Our data set included 777 neurons (*n* = 8 mice), ranging from 47 to 80 neurons (average number: 60 neurons) for each imaging plane (e.g., Fig. 1b). The activities of many neurons were simultaneously imaged during the presentation of rhythmically repeated sound stimuli, and both randomly occurring spontaneous (Fig. 1c, left) and pure tone-evoked (Fig. 1c, right) Ca^2+^ transients were observed in the imaged neurons. In addition, we performed a *post hoc* histological experiment with a retrograde neuronal tracer, CTB, which was injected into the imaged area (Fig. 1d) and we found retrogradely-labeled cortical projecting neurons mainly located in the ventral part of the lateral medial geniculate body (MGBv) (Fig. 1e), confirming that the recorded cortical site was located on the A1. This result is consistent with the connectivity between MGB and A1, as some previous studies have reported^5,44,45,50^.

### Rhythmic tone stimulation induces echo responses in the A1 L2/3 neurons

**Figure 2:**
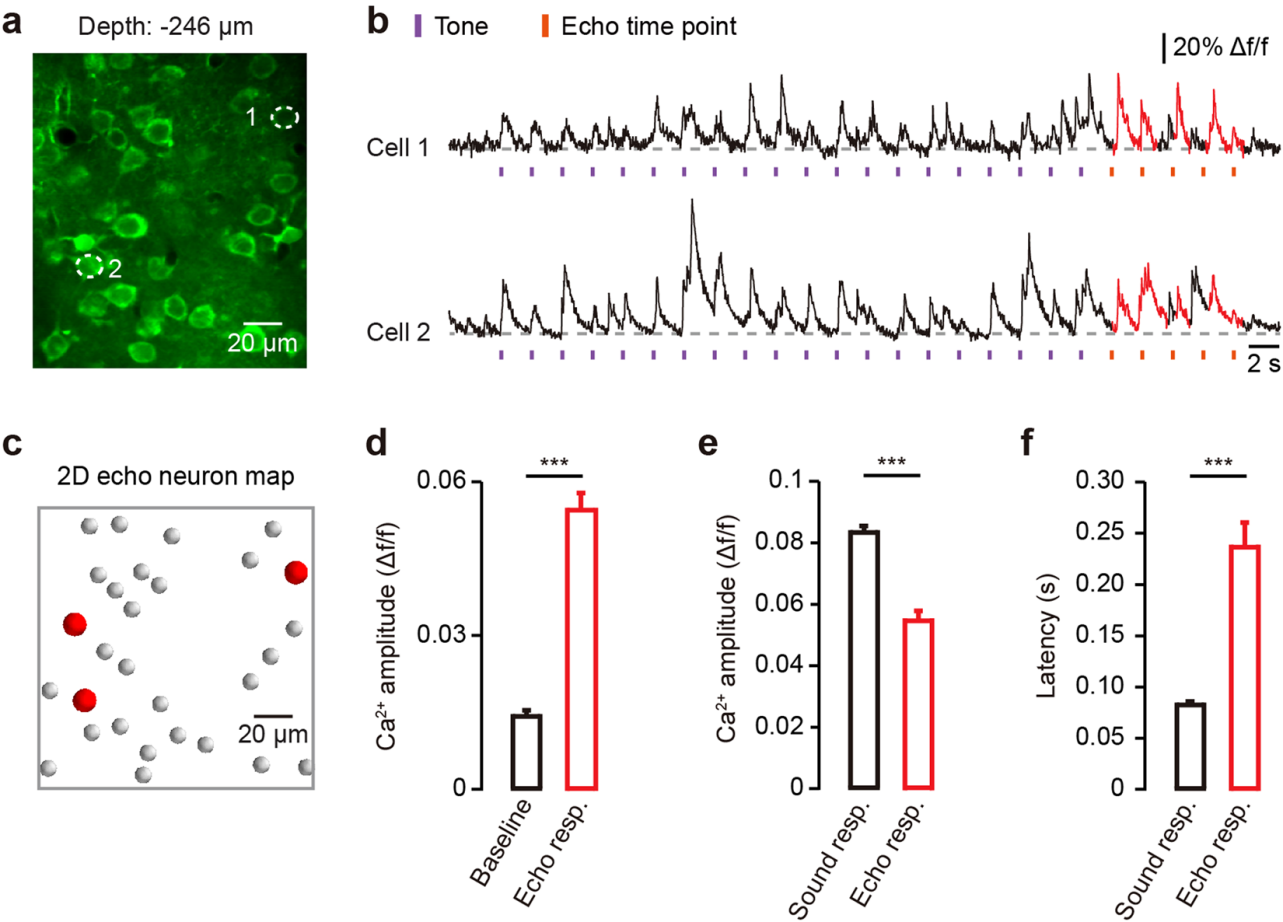
Neurons in L2/3 of A1 of anesthetized mice generate echo responses with rhythmic tone stimulation. (**a**) Two-photon image of Cal-520 AM-labeled neurons in L2/3. (**b**) Two representative neurons marked in the image of panel a show Ca^2+^ responses before, during and after tone stimuli (frequency: 30 kHz) under anesthesia. The echo responses of the neurons are marked in red. (**c**) Summary of neurons (red) that generated echo responses with rhythmic tone stimulation on this imaging plane. (**d**) The average amplitudes of echo response and baseline activity. Wilcoxon signed rank test, ***P < 0.001, *n* = 8 mice, *n* = 777 imaged neurons. (**e**) The average amplitudes of sound-evoked response and echo response. Wilcoxon rank sum test, ***P < 0.001, *n* = 8 mice, *n* = 777 imaged neurons. (**f**) The average latencies of sound-evoked response and echo response. Wilcoxon rank sum test, ***P < 0.001, *n* = 8 mice, *n* = 777 imaged neurons. Error bars indicate SEM.

To study the echo response to tonal stimulation, we presented rhythmic pure tones at each imaging plane. Figure 2a shows two representative neurons recorded in L2/3. The responses of these neurons to 20 repetitive tone stimuli (30 kHz) with an inter-stimulus-interval (ISI) of 2 s are presented in Fig. 2b. After the end of the rhythmic tone stimulation, these neurons exhibited 1–5 additional responses at the expected times for the same ISI (Fig. 2b, red), which is consistent with the neuronal echo response that has been described recently^5^. The observed echo responses were derived from a subgroup of the imaged neurons, as the neurons (Fig. 2a) represented in red (Fig. 2c). We found that 15% of the imaged neurons (15.7% ± 1.3%) showed echo responses. The average amplitude of the echo responses (0.055 ± 0.003 Δf/f) for all of the imaged neurons in L2/3 showed a significant increase comparing to that of the baseline activity (0.014 ± 0.001 Δf/f, *n* = 777 neurons from 8 mice, *P* < 0.001; Fig. 2d). When comparing the sound response with the echo response, we found the average amplitude of the sound responses (0.083 ± 0.002 Δf/f) for all of the imaged neurons in L2/3 was significantly higher than that of the echo responses (0.055 ± 0.003 Δf/f, *n* = 777 neurons from 8 mice, *P* < 0.001; Fig. 2e), and the latency of the sound responses (0.083 ± 0.001 s) was significantly shorter than that of the echo responses (0.237 ± 0.024 s, *n* = 777 neurons from 8 mice, *P* < 0.001; Fig. 2f). These results indicate that there are clear discrepancies between the echo response and the sound response with regarding to the activation strength and temporal precision.

### Frequency selectivity of echo responses in the A1 in anesthetized mice

**Figure 3:**
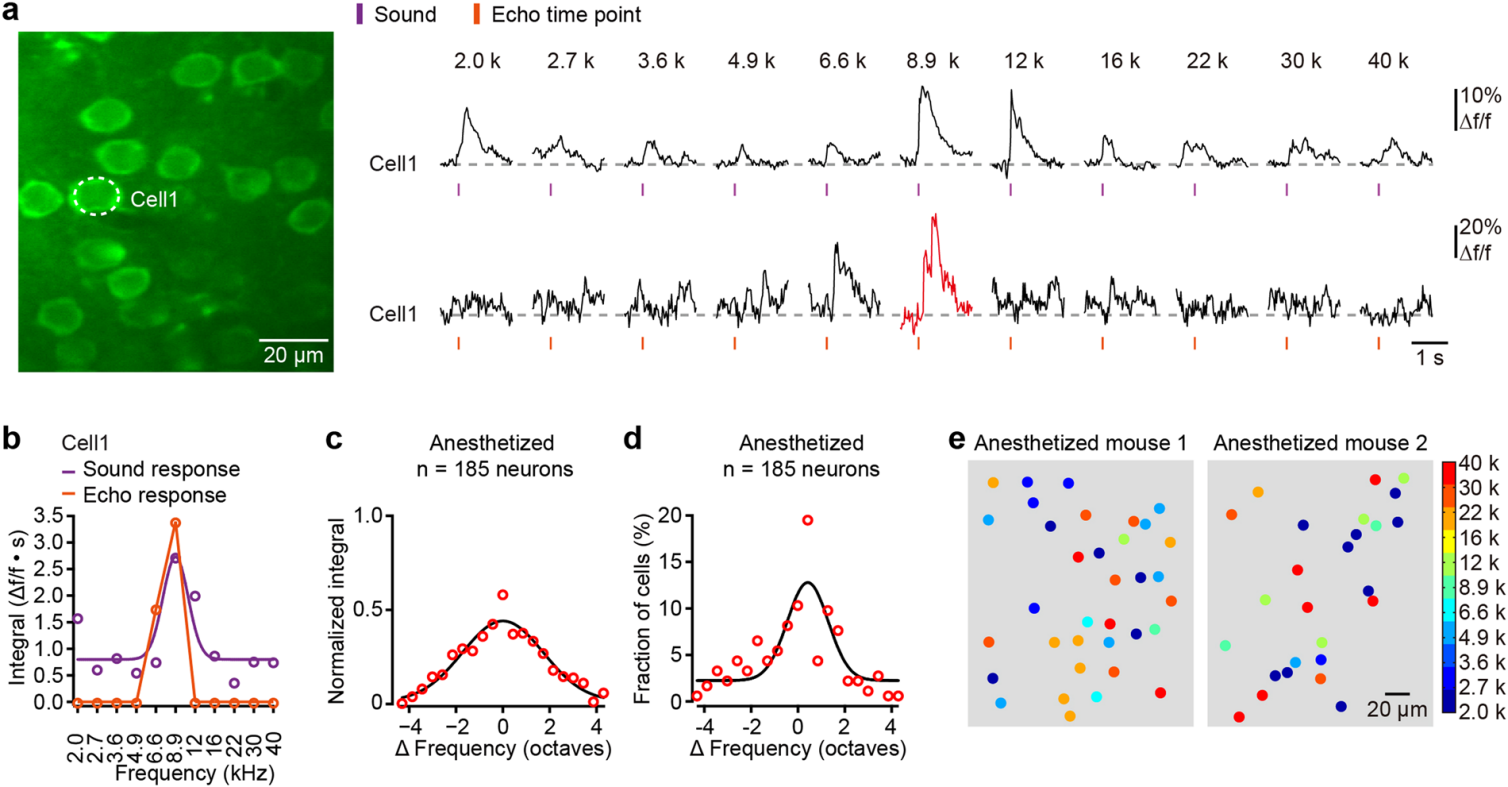
A1 neurons preferentially show echo responses to their best frequencies in anesthetized mice. (**a**) Left: two-photon imaging of neurons in L2/3; right: tone-evoked responses (upper, average of 20 trials) and echo responses (lower, single trials) in one example neuron outlined in the left panel. Eleven frequencies of pure tones were applied. Each frequency of stimulation was presented repeatedly for 20 times with an interval of 2 s. The echo response of the neuron is marked in red. (**b**) Frequency tuning of the neuron presented in panel a for the tone-evoked responses (purple) and the echo responses (orange). The points of the curves are the integrals of the Ca^2+^ responses within 500 ms (the data fitted with Gaussian function). (**c**) The average tone-evoked frequency tuning property (the data fitted with Gaussian function) of 185 echo neurons from 6 anesthetized mice. (**d**) Distribution of the difference in preferred frequency (ΔFrequency) between the tone response and the echo response (the data fitted with Gaussian function) in 6 anesthetized mice. (**e**) Spatial distribution of the echo neurons in two example imaging planes from two anesthetized mice. The frequencies are those that evoked echo responses.

To investigate whether the echo responses of the neurons were related to their frequency selectivity, we evoked echo responses with pure tones over a series of 2 to 40 kHz (every frequency was repeated 20 times with an ISI of 2 s) in anesthetized mice. Figure 3a shows an example neuron recorded in an anesthetized mouse that could be activated by sound stimulation at several frequencies, and the frequency evoked the greatest responses was 8.9 kHz. Remarkably, the echo responses were evoked in this neuron when the stimulation sequence consisted of tones close to 8.9 kHz (Fig. 3b). Although the overall tuning for sound-evoked responses across all of the echo neurons (Fig. 3c) was relatively broad given the sound level used here, the echo responses in most of the neurons occurred at the preferred frequencies. A comparison of the properties of the sound responses and the echo responses revealed that the frequency that evoked the echo response in a given neuron closely corresponded to its preferred frequency to tone stimulation in anesthetized mice (Fig. 3d). We also analyzed the spatial organization of the frequency-tuned echo neurons. Echo neurons in response to different frequencies were spatially mixed over a scale about 200 µm × 200 µm: adjacent neurons were capable of displaying echo responses to very different frequencies we employed here (Fig. 3e).

### Frequency selectivity of echo responses in the A1 in awake mice

**Figure 4:**
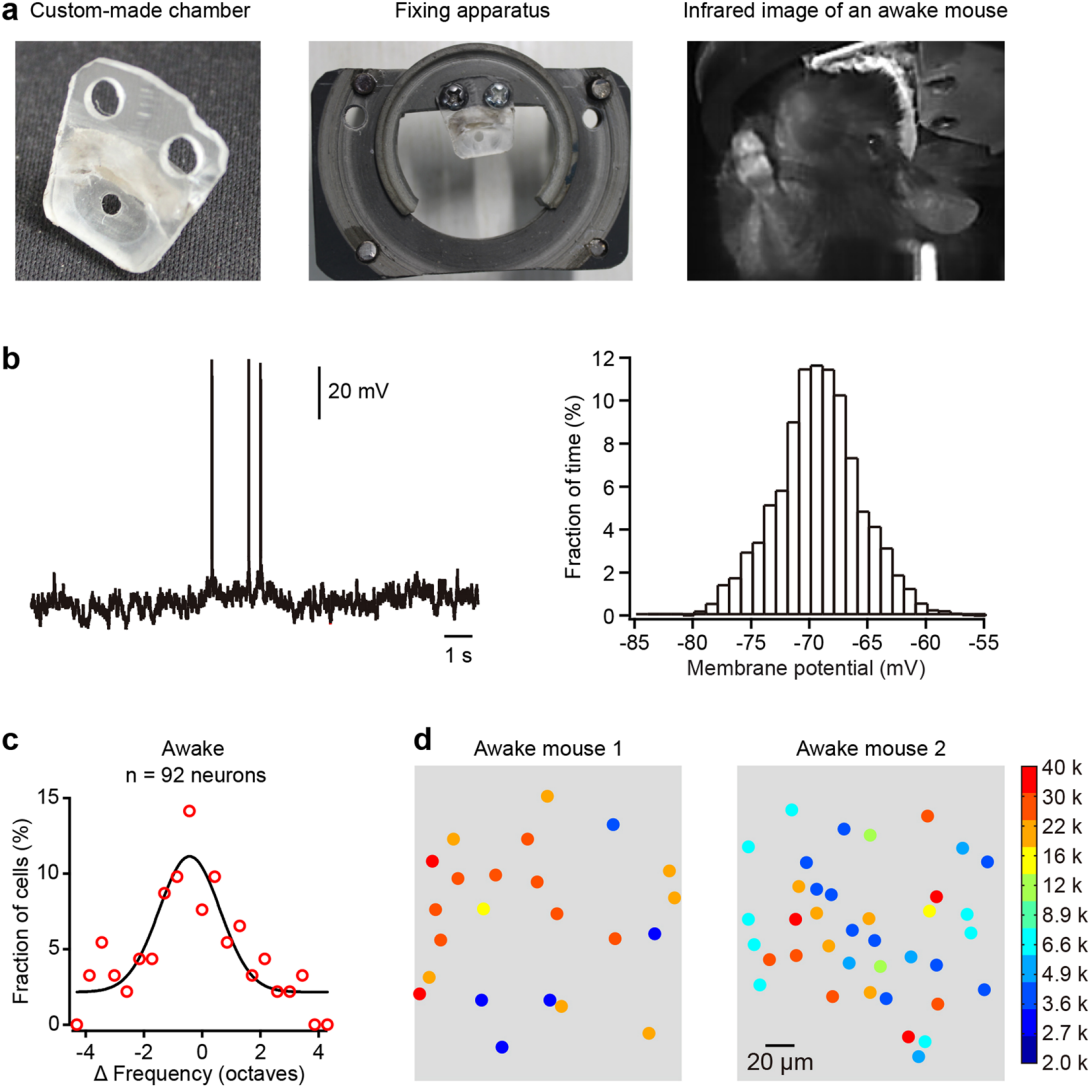
A1 neurons preferentially show echo responses to their best frequencies in awake mice. (**a**) Left and middle: a custom-made chamber for an awake mouse and its fixing apparatus; right: infrared image of a head-fixed mouse in the two-photon imaging setup. (**b**) Left: whole-cell recording in an example neuron from an awake mouse; right: the membrane potential (binned at 1 mV) distribution from the neuron presented in the left panel. (**c**) Distribution of the difference in preferred frequency (ΔFrequency) between the tone-evoked response and the echo response (the data fitted with Gaussian function) in 4 awake mice. (**d**) Spatial distribution of the echo neurons in two example imaging planes from two awake mice. The frequencies are those that evoked echo responses.

It is widely believed that general anesthesia will have an impact on lots of aspects of cortical activities. So we repeated these experiments in awake mice. We exploited a specially-made chamber and a fixing setup (Fig. 4a, left and middle), thus keeping the surface of the A1 vertical to the microscope objective. From the camera recording, we could know the mice were awake by observing the behavior such as eye opening, whisking and struggling (Fig. 4a, right). Whole-cell current-clamp recording in a L2/3 neuron of A1 showed that high-frequency but small membrane potential variations with a unimodal distribution further proved the mouse was in the awake condition (Fig. 4b). These membrane potential fluctuations differed from the slow but large fluctuations recording in anesthetized mice. We then repeated the experiments to study the frequency selectivity of echo responses in awake mice. By comparing the preferred frequencies between sound responses and echo responses (92 neurons from 4 awake mice), we found that the frequency that evoked the echo response also closely corresponded to the preferred frequency to tone stimulation in awake mice (Fig. 4c). The spatial distribution of the preferred frequency for evoking an echo response in neurons was also mixed at a small scale (Fig. 4d). Therefore, these results suggested that the echo responses of neurons within A1 are highly related to their frequency selectivity.

## Discussion

Using two-photon Ca^2+^ imaging in combination with the improved fluorescent Ca^2+^ indicator Cal-520 AM, we confirmed that neuronal responses representing predictive information can be observed in the mouse A1. The results showed that a subgroup of neurons in L2/3 exhibited echo responses at the anticipated time interval following a train of rhythmic pure tone stimulation. This is consistent with the representations of predictive sound information in the A1 reported in a previous study that used broadband noise stimulation^5^. Furthermore, we found that these echo responses are preferentially observed near the preferred frequencies of the neurons during sound stimulation. In an imaged region of approximately 200 µm × 200 µm, neurons showed echo responses to any of the tested frequencies, suggesting heterogeneous organization of mouse A1 neurons in the coding of predictive information.

**Figure 5:**
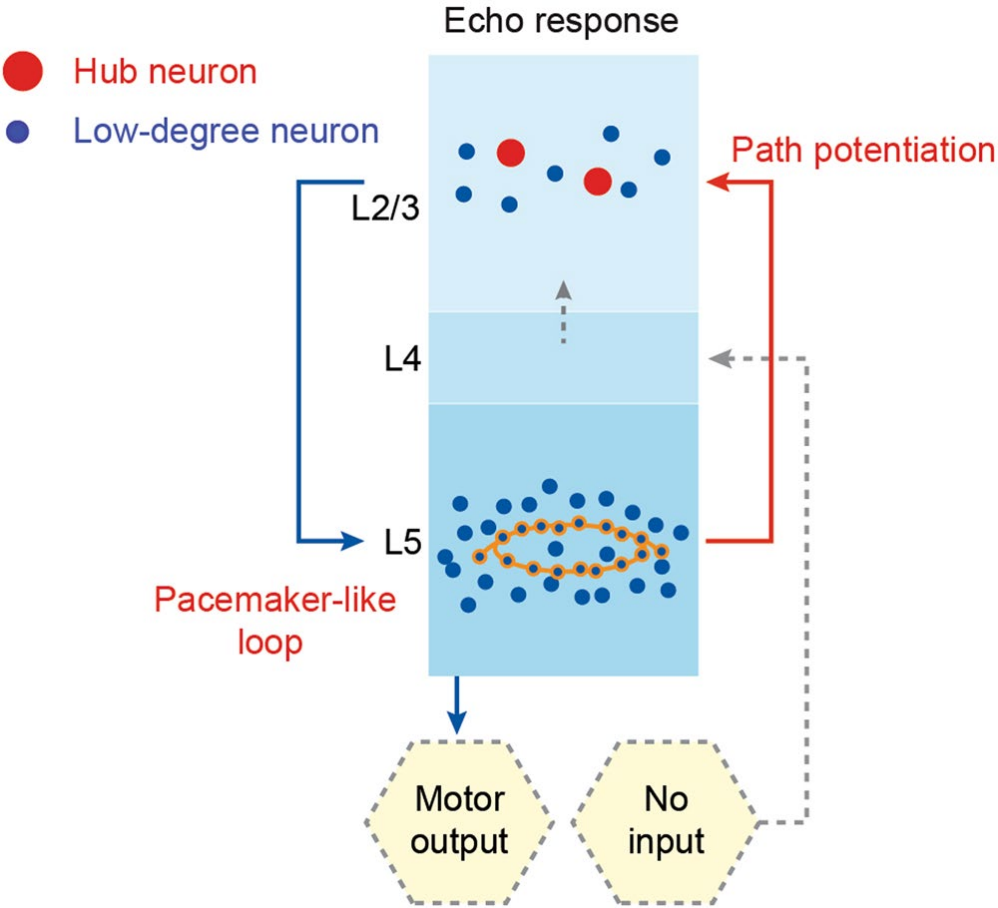
Working model for generating echo response in cortical networks. After rhythmic sensory stimulation, a loop of low-degree neurons in L5 is formed with a certain length in the neural network to match the interval of the external input rhythm. Meanwhile, the connections from L5 low-degree neurons to L2/3 hub neurons have been strengthened for transmitting signals from the selected L5 loop (pacemaker-like loop) to L2/3. Finally, the neural network is able to generate echo responses with the same period as the rhythmic input.

In previous studies, it has been shown that temporal intervals of visual inputs can be memorized by neural systems in the order of seconds^3^. Thus, understanding the mechanisms of how temporal information is processed in neural systems is crucial for elucidating brain functions^1^, such as for speech recognition and music appreciation. For the possible mechanisms of how echo responses arise, a model proposed by Mi *et al*.^51^ matches our previous^5^ and current results. The simulation of the model reproduced the experimentally observed rhythmic synchronous firing, and it provides supports for the idea that temporal information can be processed by the dynamics of distributed circuits in the neural system. In this study, we believe that this model can also be used for explaining the mechanism for generating echo response following rhythmic tone stimulation. We propose that, there is scale-free topology in the cortical network, which contains hub neurons in L2/3 and many low-degree loops with different lengths in L5 (Fig. 5; modified from the previous study^5^). These low-degree loops enable the neural system to follow the external input rhythm, while hub neurons can trigger synchronization in the whole network. During learning, the connectivity from the L5 loops to the L2/3 hubs is highly strengthened, which allows the signals to flow across the entire network. After learning through a train of rhythmic sensory stimulation, a loop with certain length is selected to match the input rhythm. Finally, the network is able to generate echo responses with the same rhythm. Therefore, it implies that echo response is an intrinsic property of cortical circuits in response to the absence of an expected sound, and echo response may reflect a distinct neural process in comparison to sound-evoked responses. In our result, we found significant differences in the amplitudes (Fig. 2e) and the latencies (Fig. 2f) between sound-evoked responses and echo responses. Those differences could be attributed to a variation in neurons for the estimation of the input rhythm, and it thus leads to a longer latency and a lower amplitude of the echo responses on average.

A presumption in neuroscience shows that a brain is capable of effectively performing genuine Bayesian inferences^52^. It proposes that predictive coding may give an explanation on how Bayesian inference could be performed in the cerebral cortex from the neural perspective^53,54^. Theoretically, predictive coding suggests that the brain is constantly making efforts to ‘fit’ internal models in perceiving through inferring the incoming sensory input. The discrepancies between the actual sensory data and the predicted sensory data (‘error’) are used to refine the model in an iterative process until the model optimized (‘error’ minimized). Our studies based on unexpectedly omitted stimuli can give support for the prospective and predictive property of the expectancy outcomes, which would suggest the echo response as possibly the signature finding of predictive coding, as it is even able to be observed when sensory input is absent. Furthermore, predictive coding theory assumes that neural responses are shaped by expectations, which are hierarchically organized. For instance, a simple anatomical assumption is that prediction and error computations are implemented by separate neurons that reside in different cortical layers^55^. However, evidences by far are still deficient in the auditory field in accordance with the notion that prediction and error neurons reside separately in distinct cortical layers^56^, which shall be studied further.

Receptive fields of neurons represent how sensory information is encoded and mapped for the guidance of perception and behavior^57^. The neurons in the A1 can form spectro-temporal filters to enhance the auditory representation of stimuli along two-feature dimensions, incorporating both the frequency and the timing information of the relevant auditory stimulus sequence^12^. For example, neurons in the A1 had consistent spectral and temporal features to maintain stable receptive fields for several hours^58^. In our results, we found that the preferred frequency of echo responses was very close to the preferred frequencies of the sound-triggered responses of the neurons in mice under both awake and anesthetic conditions. These results suggest that neurons in the A1 maintained stable spectral receptive fields to form a reliable, consistent representation of tonal information for the prediction.

The precise tonotopic organization in auditory cortex is a long debated issue^20,21,26^ and remains unresolved. On the macroscopic scale level (~1 mm), the tonotopic map in the A1 arises from MGBv. However, the frequency selectivity of neighboring neurons can be very different, and no reliable tonotopic organization could be observed over spatial scales of <200 µm^22^. Early studies have shown that tonal selectivity in cats^59^ and response variability in rats^60^ were increased under anesthesia, suggesting anesthetics would alter tonotopic map to be stricter and more regular. As neuronal dynamics are modulated by the type and depth of anesthesia, the observed topographic organization can be altered and thus there were a great number of experiments under light anesthesia conditions. Previous studies of using two-photon Ca^2+^ imaging in the anesthetized mouse brain demonstrated that the response profiles of nearby neurons were quite different within local circuit in A1^21,25^. Moreover, a recent study also showed a sound-evoked local heterogeneity map of the functional organization in the A1 in both lightly anesthetized and awake mice^32^. Similarly, in this study we found local heterogeneity of echo responses (Figs 3e and 4d) in L2/3 in both the awake mice and the isofluorane (0.4–0.8%) anesthetized mice. In our results, the frequency selectivity profiles of echo responses (Figs 3 and 4) are similar between anesthetized and awake mice, suggesting that the local heterogeneous organization for predictive coding could be observed under awake and light anesthesia conditions. However, the precise effects of anesthesia on A1 neurons of mouse are still largely unknown and hence need further investigation.

Therefore, our findings provide evidence for the heterogeneity of tonal organization for the coding of prediction in the A1. The fact that the local topographic organization in L2/3 of A1 was heterogeneous may support the hypothesis that neurons with different frequency selectivity interact to represent the predictive pattern of complex sounds^21^. However, the underlying mechanisms of contribution to behavior are still largely unknown and warrant investigation in future studies.
